# Discovery and Validation of Novel microRNA Panel for Non-Invasive Prediction of Prostate Cancer

**DOI:** 10.7759/cureus.58207

**Published:** 2024-04-13

**Authors:** Shweta Kumari, Anveshika Manoj, Sumit Rungta, Manoj Kumar, Gautam Prasad, Durgesh Kumar, Abbas A Mahdi, Mohammad Kaleem Ahmad

**Affiliations:** 1 Biochemistry, King George's Medical University, Lucknow, IND; 2 Gastroenterology, King George's Medical University, Lucknow, IND; 3 Urology, King George's Medical University, Lucknow, IND

**Keywords:** castration-resistant prostate cancer, panel mirna, diagnostic biomarker, non-invasive, microrna, prostate cancer

## Abstract

Background: Early diagnosis remains a challenge for prostate cancer (PCa) due to molecular heterogeneity. The purpose of our study was to explore the diagnostic potential of microRNA (miRNA) in both tissue and serum that may aid in the precise and early clinical diagnosis of PCa.

Materials and methods: The miRNA expression pattern analysis was carried out in 250 subjects (discovery and validation cohort). The Discovery Cohort included the control (n = 30) and PCa (n = 35) subjects, while the Validation Cohort included the healthy control (n = 60), benign prostate hyperplasia (BPH) (n = 55), PCa (n = 50), and castration-resistant PCa (CRPC) (n = 20) patients. The expression analysis of tissue (Discovery Cohort) and serum (Validation Cohort) was carried out by quantitative polymerase chain reaction (qPCR). The diagnostic biomarker potential was evaluated using receiver operating characteristics (ROC). Bioinformatic tools were used to explore and analyze miRNA target genes.

Results: MiRNA 4510 and miRNA 183 were significantly (p<0.001) upregulated and miRNA 329 was significantly (p<0.0001) downregulated in both PCa tissue and serum. ROC curve analysis showed excellent non-invasive biomarker potential of miRNA 4510 in both PCa (area under the curve (AUC) 0.984; p<0.001) and CRPC (AUC 0.944; p<0.001). The panel of serum miRNAs (miRNA 183 and miRNA 4510) designed for PCa had significant and greater AUC with both 100% sensitivity and specificity. Computational analysis shows that the maximum number of target genes are transcription factors that regulate oncogenes and tumor suppressors.

Conclusion: Based on ROC curve analysis, miRNAs 4510, 329, and 711 were identified as potential non-invasive diagnostic biomarkers in the early detection of PCa. Our findings imply that a panel of miRNAs 183 and 4510 has high specificity for distinguishing PCa from healthy controls and providing therapeutic targets for better and earlier PCa therapy.

## Introduction

Prostate cancer (PCa) accounts for the second most common cancer found in males after lung cancer in terms of mortality [1,2]. The multifocal nature of PCa and its indolent growth rate are responsible for false positive errors in diagnosis by various screening methods like serum prostate-specific antigen (PSA) level, and digital rectal examination (DRE) [3]. The treatment options for the detrimental last stages of PCa, castration-resistant PCa (CRPC) include a combination of chemotherapy and radiotherapy, which have serious health effects and increase the life expectancy only by a few years [4]. However, the patient still remains in pain. Therefore, diagnosing the disease early can avoid such trauma in the future and provide better treatment opportunities in the early phase of the disease. The rapid advancement of technology has led researchers to explore microRNA (miRNA) as molecular biomarkers to objectively screen and evaluate the stages of the disease.

miRNA is a short strand of 22-bp nucleotide that regulates genes or complex networks of genes at both transcriptional and translational levels by binding to the 3’ untranslated regions of target messenger RNA [5]. They play a vital role in many biologically important signaling pathways like PI3-Akt, Wnt, and Hippo, contributing to significant processes like cell differentiation, cell cycle, proliferation, inflammation, and signal transduction. Some of these processes such as migration, invasion, stemness, and proliferation are hallmarks of carcinogenesis. They are resistant to degradation and can be detected in biological fluids like saliva, serum, plasma, and urine. Therefore, miRNAs are extensively researched as non-invasive diagnostic biomarkers of PCa.

Recent studies suggest the dysregulation of miRNA leads to carcinogenesis. Some researchers have shown that the expression of miRNA in both tissue and blood may act as potential biomarkers for the early diagnosis of cancer [6,7]. Their expression behavior as oncogenic or tumor suppressors is dependent on the microenvironment of the tissue or organ. For example, miRNA 183 is found to be upregulated in various cancers like lung, breast, stomach, thyroid, and uterine, but its expression is downregulated in cervical cancer [8]. MiRNA 711 is reported to have significantly upregulated expression in head and neck cancer and urinary bladder cancer [9]. In our previous study, Waseem et al. carried out microarrays among adjacent healthy tissue and PCa tissue to explore various differentially expressed miRNAs, including miRNA 183-5p, miRNA 4510, miRNA 711, and miRNA 329-3p [10]. In the current study, we are going to validate the expression of miRNA 183-5p, miRNA 4510, miRNA 711, and miRNA 329-3p in both tissue and serum of PCa and investigate their non-invasive biomarker potential as individual miRNAs as well as a panel of circulating miRNAs.

## Materials and methods

Patient characteristics and sample collection

The patients were selected from the Department of Urology, King George's Medical University, Lucknow, India. The ethical framework for sample collection was approved by the Institutional Ethical Committee (IEC) (96th ECM IIA/P10), at King George's Medical University. Informed voluntary and written consents were acquired for cases and controls recruited in the discovery and validation cohort.

**Figure 1:**
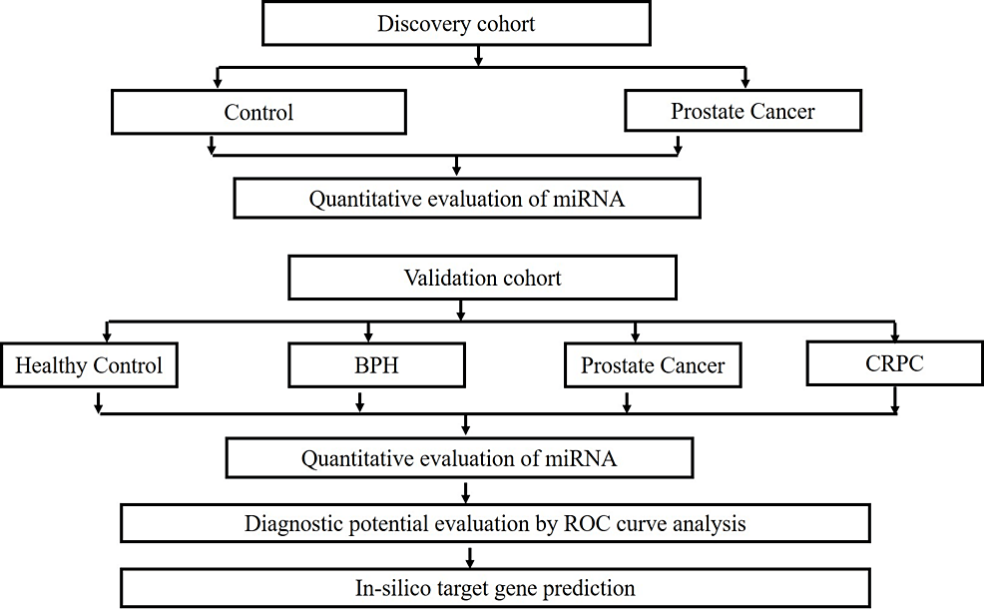
Flow chart representing the study design. BPH: benign prostate hyperplasia; CRPC: castration-resistant prostate cancer; miRNA: microRNA; ROC: receiver operating characteristic

The patients were divided into two cohorts. The Discovery Cohort included the control and PCa (case) subjects, while the Validation Cohort included healthy control, BPH, PCa, and CRPC patients. For tissue sample collection in the Discovery Cohort, the inclusion criteria were subjects above 40 years undergoing either transrectal ultrasonography (TRUS)-guided biopsy or transurethral resection of the prostate (TURP). The tissue samples were sent for histopathology for calculation of Gleason Score to differentiate cases from controls. The blood samples for the validation cohort were collected from BPH (n=55), PCa (n=50), CRPC (n=20), and healthy males (n=60) from the Department of Urology, King George's Medical University (Figure 1). The blood samples of healthy controls were collected from patients with either urinary tract infection or inflammation of the prostate gland. For BPH and PCa, the samples were collected based on the Gleason score. Lastly, blood samples of CRPC cases were taken if they had rising PSA levels after androgen depletion therapy (ADT) or undergoing any chemotherapy. Those cases unwilling to participate in the study, below 40 years of age, or suffering from any other cancer were excluded from the study. The tissue samples were preserved in RNAlater® (Thermo Fisher Scientific Inc., Waltham, Massachusetts, United States) and stored at -80°C. The whole blood was collected in plain vials. Serum was isolated from whole blood and stored in Qiazol reagent (QIAGEN N.V., Hilden, Germany) at -80°C for miRNA isolation [6]. The PSA levels were recorded for the PCa group in both cohorts. According to D’Amico’s risk classification, a PSA level above 20ng/ml was considered a high risk of PCa.

MicroRNA isolation

The serum samples stored in Qiazol were used to isolate the circulating miRNA. The process of isolation was carried out by miRNAeasy serum/plasma kit (QIAGEN), and the miRNA in tissue samples was isolated using mirVana™ miRNA Isolation Kit (Thermo Fisher Scientific Inc.). The miRNA isolated was converted into cDNA using MiRNA-X miRNA First-Strand Synthesis Kit (Takara Bio Inc., Kusatsu, Shiga, Japan) as per the manufacturer’s recommendation, and stored at -80°C, for future analysis.

Expression analysis by qPCR

In order to validate the expression of miRNA in both tissue and serum, we performed quantitative polymerase chain reaction (qPCR) using cDNA as a template, forward primers (Table 1), and SYBR Green in the TB Green Advantage® qPCR Premix (Takara Bio, Inc.). The reverse primer is a universal primer provided by TB Green Advantage qPCR Premix. The reaction volume was 25 µl and the reaction conditions were denaturation 95ºC, 10 second qPCR, x 40 cycles 95ºC five seconds, 60ºC 20 seconds, and dissociation curve 95ºC 60 seconds, 55ºC 30 seconds, and 95ºC 30 seconds. The expression of miRNA was normalized using U6 as endogenous control. Later for the interpretation of data, the Ct values of each sample for every miRNA and U6 were used for calculating the fold change (2-ddCt) expression values.

**Table 1:** Primers for qPCR qPCR: quantitative polymerase chain reaction

S. No.	MicroRNA	Accession ID	Primer
1	183-5p	MIMAT0000261	TATGGCACTGGTAGAATTCACT
2	4510	MIMAT0019047	TGAGGGAGTAGGATGTATGGTT
3	711	MIMAT0012734	GGGACCCAGGGAGAGACGTAAG
4	329-3p	MIMAT0001629	AACACACCTGGTTAACCTCTTT

In silico tools

Various computational tools for in silco work were used for exploring the target genes of each miRNA. The target gene prediction software includes TargetScan (Whitehead Institute for Biomedical Research, Cambridge, Massachusetts, United States), starBase v2.0 (http://starbase.sysu.edu.cn/), and miRDB (https://mirdb.org/). The genes were selected based on their context score, rank, seed sequence, etc. The selected target genes were categorized according to their biological process, molecular function, and the pathways they are involved in. The categorization was done by Database for Annotation, Visualization and Integrated Discovery (DAVID) 6.8 (National Institutes of Health, Bethesda, Maryland, United States) followed by Kyoto Encyclopedia of Genes and Genomes (KEGG) (Kanehisa Laboratories, Kyoto, Japan) and Reactome (https://reactome.org). The target genes were clustered into oncogene, tumor-suppressor, transcription factors etc., by Gene Set Enrichment Analysis (GSEA). 

Statistical analysis

The expression data were statistically analyzed with IBM SPSS Statistics for Windows, Version 21.0 (Released 2012; IBM Corp., Armonk, New York, United States) and GraphPad Prism 5.0 (Dotmatics, Boston, Massachusetts, United States). We used the Mann-Whitney U and Wilcoxsen Rank test to analyze non-parametric expression data. The area under the curve (AUC), sensitivity, and specificity of the biomarkers for CRPC, BPH, and PCa were calculated using Youden's index employing receiver operating characteristic (ROC) curves. A significant result was defined as p<0.05 and highly significant as p<0.001.

## Results

Patient characteristics

In the present study, we have enrolled 65 subjects in the Discovery Cohort and 185 study subjects, including 60 control subjects, 55 subjects undergoing TURP as BPH, 50 subjects undergoing TRUS as PCa, and 20 CRPC subjects irresponsive to ADT therapy in the Validation Cohort. The clinicopathological and biochemical parameters are described in Table 2.

**Table 2:** Patient characteristics of the study. Low PSA level: <20ng/ml; High PSA level: >20ng/ml. ^a^ low risk (GS 6/7 (3 + 4) and TNM stage pT2); ^b^ intermediate (GS (4 + 3) and TNM stage pT2 or pT3a); ^c^ high (GS 8/9 and/or TNM stage pT3b/4a). PSA: prostate-specific antigen; CRPC: castration-resistant prostate cancer; TNM: tumour, node, metastasis

Clinical Characteristics		Discovery Cohort		Validation Cohort			
		Prostate Cancer	Control	CRPC	Prostate Cancer	Benign Prostate Hyperplasia	Control
		(n=35)	(n=30)	(n=20)	(n=50)	(n=55)	(n=60)
Age (years)	Mean	66.5	53.4	72.6	58.5	48.4	41.6
	Range	51-70	40-65	70-81	51-70	40-65	40-50
PSA (ng/ml)							
	Low PSA	9 (25.7%)	22 (73.3%)	0	14 (28%)	52 (94.5%)	60 (100%)
	High PSA	26 (74.3%)	8 (26.7%)	20	36 (72%)	3 (5.5%)	0
Gleason Score							
	7	12 (34.3%)		0	22 (44%)	-	-
	≥7	23 (65.7%)		20 (100%)	28 (56%)	-	-
Risk Assessment							
	Low ^a^	8 (22.8%)		0	10 (20%)	-	-
	Intermediate ^b^	15 (42.9%)		0	23 (46%)	-	-
	High ^c^	12 (34.3%)		20 (100%)	17 (34%)	-	-

Quantitative expression analysis of miRNA

**Figure 2:**
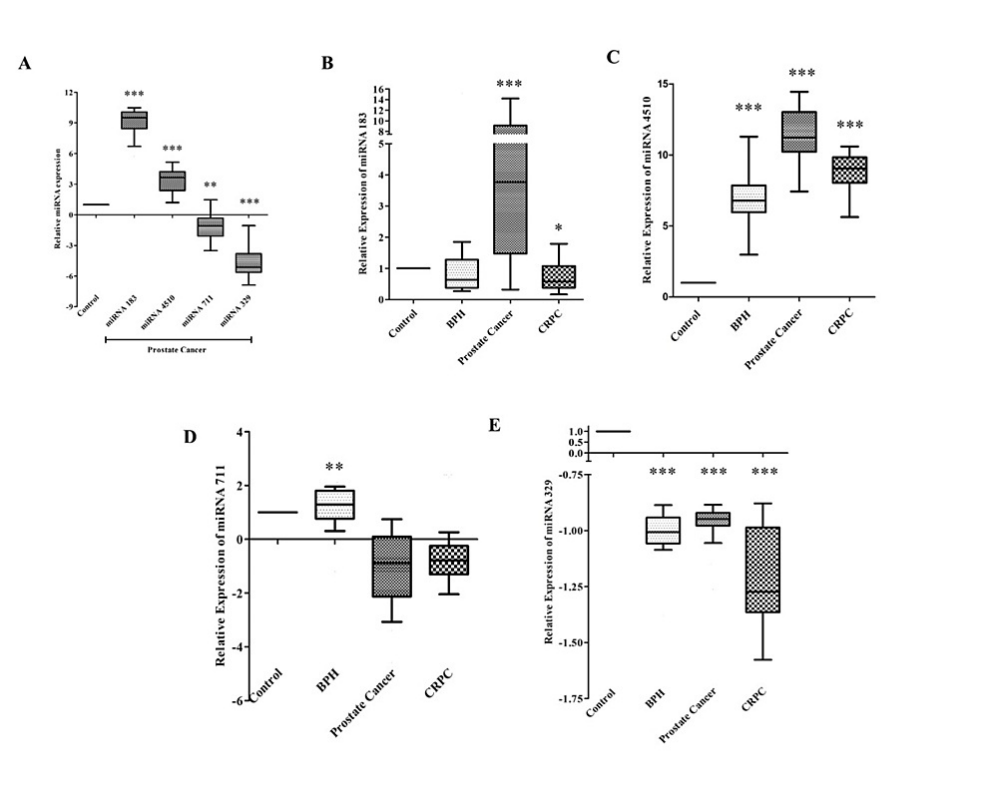
The relative expression of (A) all miRNA in prostate tissue as compared with control and the expression of (B) miRNA 183, (C) miRNA 4510, (D) miRNA 711, and (E) miRNA 329 in serum. The graph is presented in the box plot with significant values marked according to p-value: * p<0.05; ** p<0.01; *** p<0.001 miRNA: microRNA; BPH: benign prostate hyperplasia; CRPC: castration-resistant prostate cancer

Among all the miRNAs, miRNA 183 and miRNA 4510 were highly significant and upregulated in PCa tissues compared to Control tissues. On the contrary, miRNA 711 and miRNA 329 were significantly down-regulated in PCa tissue as compared with control (Figure 2A).

For the serum samples, the expression of miRNA 183 was upregulated in PCa (p< 0.001) and slightly downregulated in CRPC (p<0.05) as quantified by comparing with healthy controls (Figure 2B). Similarly, the expression of miRNA 4510 was found to be highly significant and upregulated in BPH, PCa, and CRPC groups as compared to healthy controls (Figure 2C) while the expression of miRNA 711 was insignificant and downregulated in PCa and CRPC (Figure 2D). The miRNA 329 expression in the serum of all case groups was highly significant and downregulated as compared to controls (Figure 2E).

The Wilcoxon signed-rank test carried out between serum and tissue of PCa demonstrates that among all the miRNA, the expression of miRNA 183 was highly significant and upregulated in tissue as compared to serum PCa while the expression of miRNA 4510 and miRNA 329 were highly significant and downregulated in tissues as compared to serum PCa. On the other hand, it was seen that miRNA 183 and miRNA 329 were highly significant and downregulated in tissues as compared to serum BPH. On the contrary, the expression of miRNA 4510 in the BPH group was found to be highly significant and upregulated in tissues compared to serum (Table 3).

**Table 3:** Upregulation/down-regulation of miRNA between serum and tissue. z and p values from the Wilcoxon rank sum test. Bold values are statistically significant values. The  value of p <0.05 and p<0.001 are considered signifcant.  z value is calculated as mean difference between serum and tissue expression values. miRNA: microRNA; BPH: benign prostate hyperplasia

miRNA	Prostate Cancer		BPH	
	z	p value	z	p value
183-5p	3.898	<0.001	-2.608	<0.05
4510	-5.511	<0.001	5.497	<0.001
711	-.941	0.347	0.403	0.687
329-3p	-5.714	<0.001	-5.511	<0.001

Relation of miRNA with clinicopathological parameters

Among all the miRNAs, the expression of miRNA 329 in PCa tissue and miRNA 4510 in PCa serum was found to be significantly correlated. MiRNA 329 was negatively correlated with age and miRNA 4510 was positively correlated with the Gleason score (Table 4).

**Table 4:** Association of clinicopathological parameters with miRNA expression. **p<0.05, significant. The reference PSA level ranges from 0 ng/ml to 4 ng/ml. miRNA: microRNA; PSA: prostate specific antigen

Group	miRNA	Gleason score	PSA level (ng/ml)	Age
Prostate Cancer tissue	183-5p	0.0115	-0.0018	0.0246
	4510	-0.1871	-0.0739	0.0802
	711	0.0808	-0.2646	-0.0505
	329-3p	-0.1574	0.0490	-0.3200**
Prostate Cancer serum	183-5p	0.1687	0.1741	0.0780
	4510	0.5368**	0.0822	-0.2703
	711	-0.1900	-0.2265	-0.0848
	329-3p	-0.2535	-0.1422	-0.0502

Biomarker potential of microRNA

**Figure 3:**
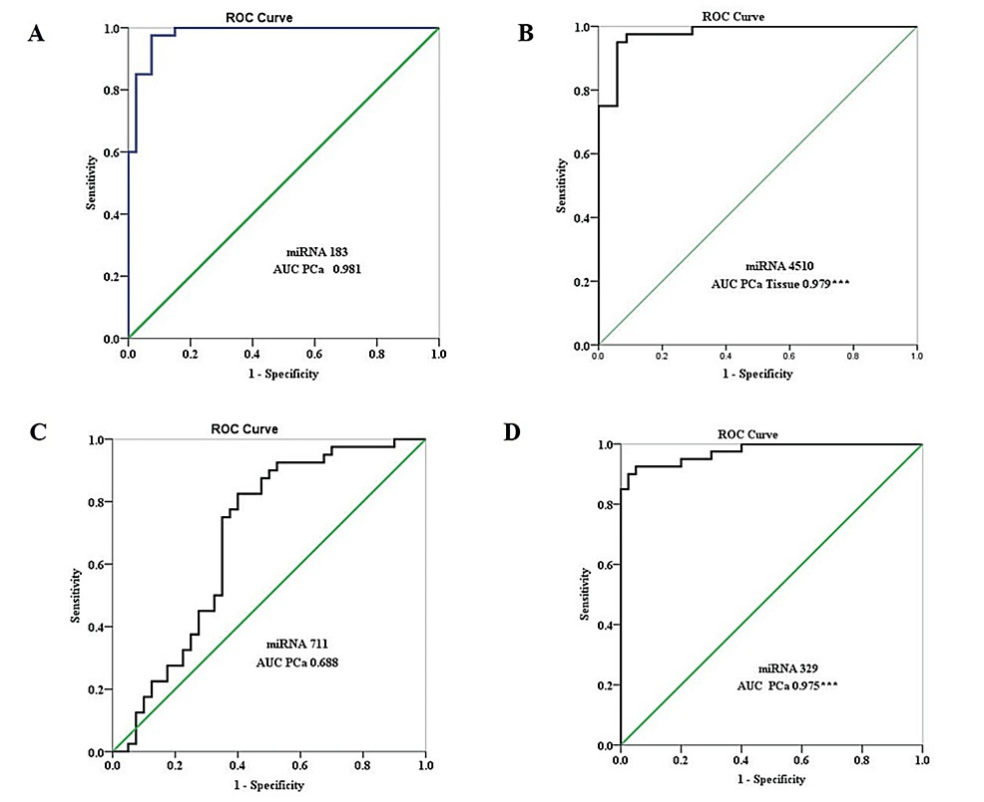
The ROC curve analysis of (A) miRNA 183, (B) miRNA 4510, (C) miRNA 711, and (D) miRNA 329 in tissue. The AUC is marked with significant values marked according to the p-value. * p<0.05; ** p<0.01; *** p<0.001 miRNA: microRNA; AUC: area under the curve

The ROC curve was plotted between sensitivity and specificity to determine the biomarker potential of miRNA in both tissue and serum. The ROC curves of individual miRNA for tissue indicate that miRNA 183, miRNA 4510, and miRNA 329 have great and significant AUC of 0.981, 0.979, and 0.975, respectively (Table 5, Figure 3). The Discovery Cohort had a significant AUC of 0.607 with 99% sensitivity and 46% specificity, combining all the miRNA as a panel (Table 5).

**Table 5:** Receiver operating characteristics of the Discovery and Validation cohorts. * p<0.05; ** p<0.01; *** p<0.001 AUC: area under the curve; CI: confidence interval; miRNA: microRNA

Parameter	miRNA	AUC	95%CI	Sensitivity %	Specificity %	Cutoff Value	Yoden's Index
Prostate Cancer Tissue	Discovery Cohort						
	miRNA 183	0.981	0.956-1.000	97.50%	92.50%	6.59	0.9
	miRNA 4510	0.979***	0.953-1.000	95%	94.10%	9.91	0.891
	miRNA 711	0.688	0.565-0.808	82.50%	60%	-0.153	0.425
	miRNA 329	0.975***	0.946-1.000	92.50%	95%	-5.09	0.875
	miRNA 183+miRNA 4510+miRNA 711+miRNA 329	0.607**	0.539-0.674	99%	46%	4.37	0.45
	Validation Cohort						
Serum Prostate Cancer	miRNA 183	0.641	0.437-0.845	45%	87.50%	2.13	0.325
	miRNA 4510	0.984***	0.956-1.000	92.50%	100%	7.97	0.925
	miRNA 711	0.693**	0.493-0.892	89.20%	50%	-1.85	0.392
	miRNA 329	0.753**	0.456-1.000	100%	75%	-3.401	0.75
	miRNA 183+miRNA 4510+miRNA 711+miRNA 329	0.645*	0.547-0.743	93.80%	68.70%	0.52	0.25
Serum Benign Prostate Hyperplasia	miRNA 183	0.634	0.404-0.865	80%	50%	0.54	0.3
	miRNA 4510	0.788**	0.573-1.000	87.50%	75%	5.029	0.625
	miRNA 711	0.206*	0.035-0.377	100%	0%	3.88	0.027
	miRNA 329	0.763**	0.456-1.000	100%	75%	0.411	0.75
	miRNA 183+miRNA 4510+miRNA 711+miRNA 329	0.45	0.332-0.567	65.60%	43.40%	-1.1	0.09
Serum Castration Resistant Prostate Cancer	miRNA 183	0.728*	0.498-0.958	97.50%	50%	2.493	0.475
	miRNA 4510	0.944***	0.866-1.000	77.50%	100%	7.97	0.775
	miRNA 711	0.693***	0.453-0.932	94.60%	50%	-1.98	0.446
	miRNA 329	0.872***	0.710-1.000	100%	75%	-0.027	0.75
	miRNA 183+miRNA 4510+miRNA 711+miRNA 329	0.701***	0.601-0.802	69.40%	62.50%	1.96	0.319

**Figure 4:**
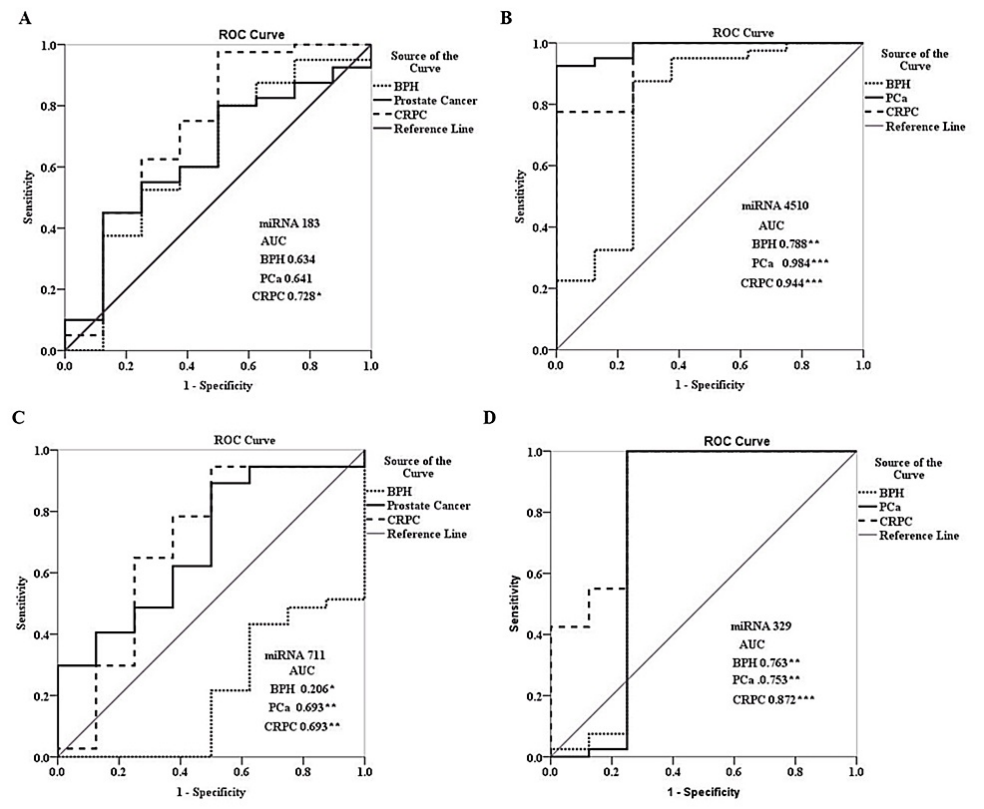
The ROC curve analysis of (A) miRNA 183, (B) miRNA 4510, (C) miRNA 711, and (D) miRNA 329 in serum The AUC is marked with significant values marked according to the p-value. * p<0.05; ** p<0.01; *** p<0.001 miRNA: microRNA; BPH: benign prostate hyperplasia; PCa: prostate cancer; CRPC: castration-resistant prostate cancer; AUC: area under the curve

The ROC curve for miRNA 183 in serum revealed greater and significant AUC (0.728) with 97.5% sensitivity and 50% specificity only for the CRPC group. The miRNA 4510 had all three groups, BPH, PCa, and CRPC, with greater and significant AUC of 0.768, 0.984, and 0.944, respectively with good sensitivity and specificity. Next, miRNA 711 had ROC greater and significant AUC of 0.693 for both PCa and CRPC groups. However, the BPH group had a significant AUC but the area covered was much lesser. Last, miRNA 329 had all three groups BPH, PCa, and CRPC with greater and significant AUC of 0.763, 0.753, and 0.872, respectively, with good sensitivity and specificity. The validation cohort of all the combined miRNA is reported with significant AUC of 0.645 and 0.701 in the PCa and CRPC groups, respectively. Serum PCa group had 93.8% sensitivity and serum CRPC group had 69.4% sensitivity (Table 5, Figure 4).

**Figure 5:**
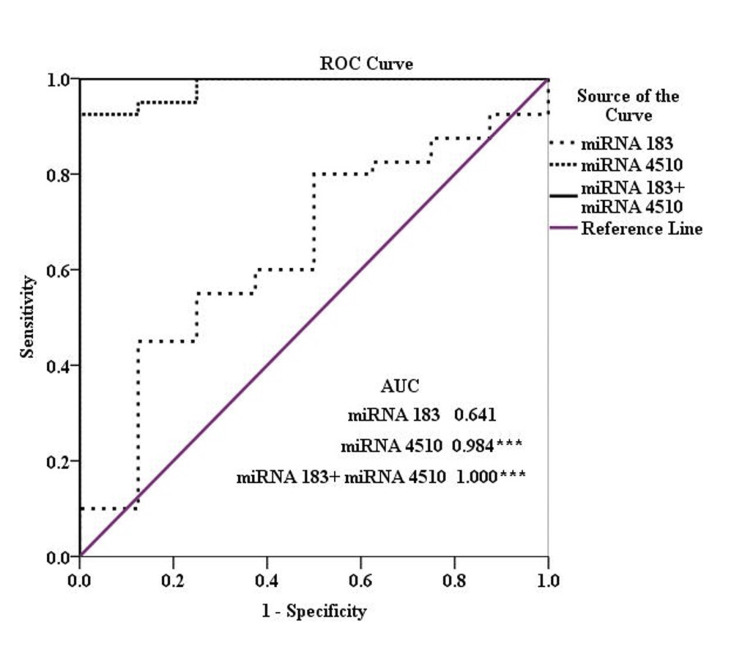
The ROC curve analysis of panel miRNA, miRNA 183 and miRNA 4510, in serum PCa group. The AUC is marked with significant values marked according to the p-value. *** p<0.001 miRNA: microRNA; AUC: area under the curve

**Figure 6:**
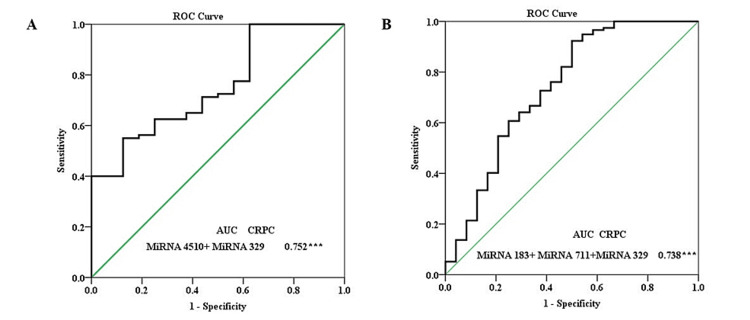
The ROC curve analysis of (A) panel miRNA 4510 and miRNA 329, (B) panel miRNA 183, miRNA 711, and miRNA 329 in CRPC. miRNA: microRNA; CRPC: castration-resistant prostate cancer; ROC: receiver operating characteristic

However, in the serum PCa group, miRNA 183 and miRNA 4510 panels covered greater and significant (p=0.008) AUC of 1.000 with 100% specificity and 100% specificity with 4.59 cutoff value at maximum Yoden's index (Figure 5). The other panels designed for CRPC are shown in Figure 6.

In silico analysis of miRNA-target gene

**Figure 7:**
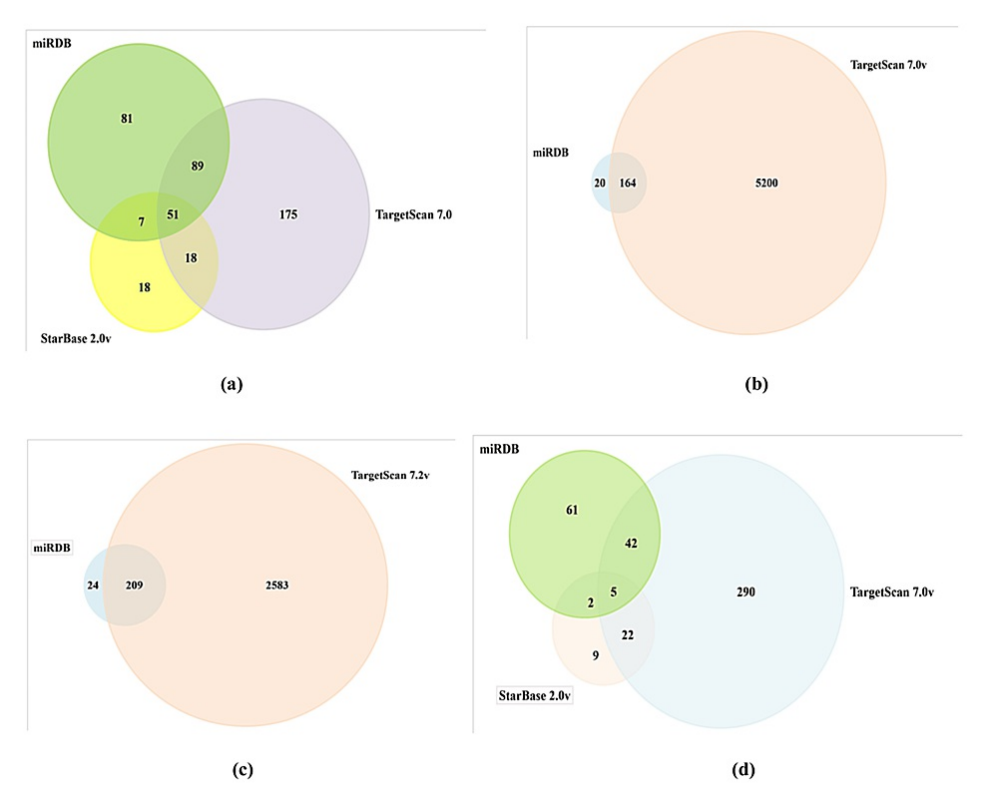
The Venn Diagram of overlapping target genes of (a) miRNA 183, (b) miRNA 4510, (c) miRNA 711, and (d) miRNA 329

**Figure 8:**
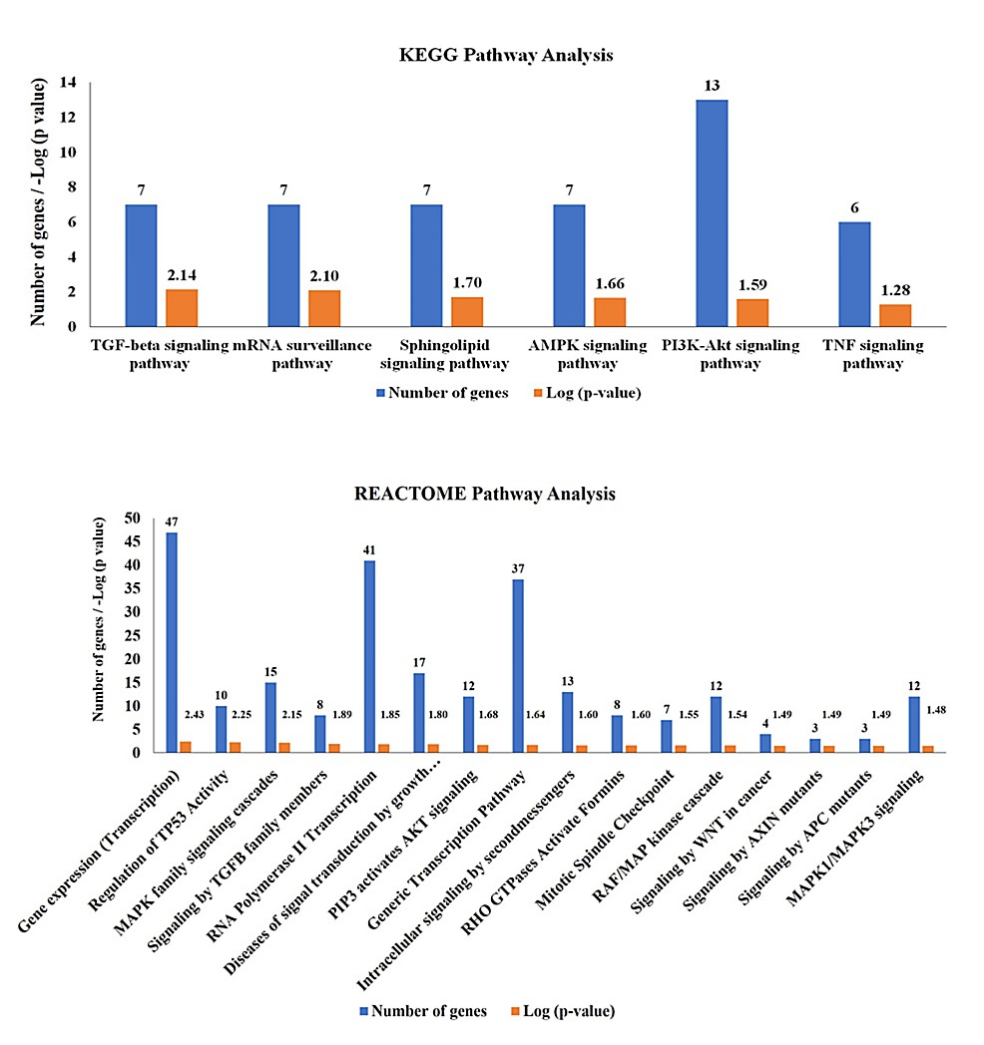
The KEGG and REACTOME pathway analysis of overlapped target genes with significant p values (log values). The target genes are selected on the basis of p value <0.05. KEGG: Kyoto Encyclopedia of Genes and Genomes Genome

**Figure 9:**
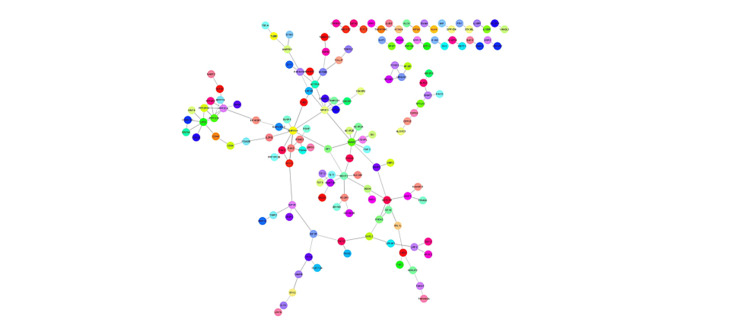
The visual representation of miRNA-Target genes by Cytoscape 3.9.1* *Cytoscape Consortium, San Diego, California, United States

The in silico analysis of genes targeted by miRNA was followed up to suggest the role of miRNA in the complex network of pathways that are involved in PCa progression and development. The target genes for each miRNA from all three in silco tools were selected according to the criterion set and Venn diagrams of overlapped target genes of each miRNA were constructed (Figure 7). In total, 429 overlapped target genes were used further to determine their KEGG pathway analysis and Reactome pathway analysis. The KEGG pathway analysis suggested that a maximum and significant number of genes were involved in the PI3-Akt signaling pathway, followed by TGF-beta, AMPK, and TNF signaling pathway (Figure 7). Similarly, Reactome pathway analysis reveals integrated molecular interactions and network data information. They were followed by RAF/MAP kinase cascade, regulation of TP53, and PIP3 activation Akt signaling (Figure 8). Among the target genes, DLX6, SP1, GATA2, FOXN2, and SND1 were transcription factors that promote gene expression of downstream and upstream target genes including oncogenes (TCF12, GATA2, LASP1), tumor suppressor genes (TET2), protein kinases (IGFR1, PAK2, PAK3, GSK3A, MAPK14), etc. (Figure 9).

## Discussion

Exploring the potential of miRNA in PCa as diagnostic and prognostic markers and leading them to therapeutic targets for better treatment and early diagnosis of PCa, is currently a dynamic field of research. Considering miRNA irresistibility to RNase and DNase and involvement in partial or complete binding to mRNA to regulate protein synthesis, makes them more suitable as biomarkers. In the present study, we have evaluated the expression of miRNAs 183, 4510, 711, and 329 in both tissue and serum of PCa as well as analyzed their biomarker potential individually in both tissue and serum of PCa. We have reported highly significant upregulated expression of miRNA 183 and 4510 and downregulated expression of miRNA 711 and 329 in PCa tissue and serum. Moreover, the panel of serum miRNA designed for PCa was analysed for the first time in our study.

The miRNA 183 is a part of the highly conserved miRNA 183-96-182 cluster located at the 7q31-34 human chromosome. They are majorly involved in the development of the retina and cochlea. Our study reported their significant upregulation in PCa tissue with highly significant and greater AUC, with 97.5% sensitivity and 92.5% specificity. However, the Wilcoxon test evaluated between the PCa serum and tissue shows that miRNA 183 expression was highly significant and upregulated in tissue by 3.89-fold as compared to the serum. However, the dysregulation of miRNA is reported to be responsible for carcinogenesis in various cancers like ovarian, esophageal squamous cell, bladder, colorectal, breast, brain glioma, colon, and prostate, with upregulated expression, while other melanoma, kidney, cervical, osteosarcoma, and gastric cancers have downregulated expression [11,12]. Despite being reported as a biomarker in PCa, there are very few studies that indicate its circulating non-invasive biomarker potential in PCa biofluids. In our study, miRNA 183 showed a 5.94-fold increase in the serum PCa group as compared to the control. Numerous studies have reported the functional role of miRNA 183 in cell migration, invasion, epithelial-mesenchymal transition (EMT), and micro angiogenesis due to the presence of binding sites of target genes like ZEB1, TGF-β, p53, PDCD4, ITGB1, and EGR1 [12-14].

The miRNA 4510 is located on chromosome 15 and is found to be significantly upregulated in PCa and CRPC in the current study as compared to healthy males. Our study reports its oncogenic behavior for the first time in PCa tissue and serum, as well as its potential biomarker ability to distinguish PCa and CRPC from healthy males. The Wilcoxon test shows that its expression in PCa serum is higher than that in PCa tissue and is significantly and positively correlated with the Gleason score. Thus, as the disease progresses with increasing aggressiveness and Gleason score, the expression of miRNA 4510 also increases in PCa serum. Its oncogenic activity has been reported in stage IA lung adenocarcinoma and ovarian cancer by Notch3 targeting [15,16]. There have been several studies to report its downregulation in various other cancers like colorectal, recurrent breast, bladder, and hepatocellular carcinoma [17-19]. One of the recent studies has indicated its involvement in the progression of liver cancer by targeting the RAF1 and RAS-ERK signaling cascades [20]. MiRNA 4510 has been reported to be involved in migration, invasion, and proliferation in gastrointestinal stromal tumors by targeting APOC2 expression [21].

This is the first time we are exploring miRNA 711 expression in BPH, PCa, and CRPC serum and comparing them with healthy males to determine its non-invasive biomarker potential. We found that serum miRNA 711 was downregulated in both PCa and CRPC. Our previous study, Waseem et al. (2017) [10], showed the downregulation of miRNA 711 in PCa tissue as compared to adjacent healthy tissue. Some studies have accounted for predicted and validated gene targets like GATA2, SKI, NTRK3, PAK2, AR, SP1, and CDK4 that describe their involvement in cancer progression [22]. It is reported to be upregulated in other cancers like head and neck, breast, cutaneous T-cell lymphoma, and urinary bladder [23,24].

The current study seems to be the first to investigate the expression of miRNA 329 in PCa serum and tissue and CRPC and report its significant tumor suppressor activity as compared with healthy males. There are very few studies exploring its relationship with PCa. Wilcoxon test showed that its expression in serum is higher than in tissue and it is significantly and negatively correlated with age. Therefore, with progressing age and increasing incidence of disease, the expression of miRNA 329 is decreased. It has shown its downregulated expression in other cancers like cervical cancer by directly targeting MAPK1, neuroblastoma tumor tissue, gastric cancer by directly targeting TIAM1, colorectal cancer by targeting TGF-β/Smad pathway, thyroid cancer by targeting WNT1, and pancreatic cancer by targeting grb2 [25-29]. Similarly, our study also predicts target genes like E2F1, WNT1, RALA, and PTEN that reveal their involvement in cancer progression.

Numerous studies based on the early detection of urological malignancies like bladder cancer, and PCa are extensively in research to provide valuable data for better management of the disease [30]. The current study provides an additional understanding of the early detection of PCa by miRNA as a diagnostic biomarker for better precise treatment. The limitation of our study is that we could not explore the prognostic potential of miRNA 4510, miRNA 183, and miRNA 329 and their functional role in the progression of PCa. Further, clinical investigation of the panel of miRNAs in PCa and CRPC groups for their diagnostic potential on large-scale samples should be carried out before an actual clinical test could be developed.

## Conclusions

The current study reports the excellent non-invasive diagnostic biomarker potential of miRNA 4510 to predict for the first time in both PCa and CRPC from healthy individuals. To the best of our knowledge, this study indicates the non-invasive diagnostic potential of miRNA 329 and miRNA 711 in differentiating PCa from healthy controls. The panel of miRNA (miRNA 183+miRNA 4510) indicates the highest specificity for early diagnosis of PCa, non-invasively. Our work lays the groundwork for clinical investigation on a larger sample size to implement this panel of miRNA as the clinical test in the future. This may help in early diagnosis of PCa and miRNA-mRNA interaction would provide novel therapeutic targets.
